# Strong selection during the last millennium for African ancestry in the admixed population of Madagascar

**DOI:** 10.1038/s41467-018-03342-5

**Published:** 2018-03-02

**Authors:** Denis Pierron, Margit Heiske, Harilanto Razafindrazaka, Veronica Pereda-loth, Jazmin Sanchez, Omar Alva, Amal Arachiche, Anne Boland, Robert Olaso, Jean-Francois Deleuze, Francois-Xavier Ricaut, Jean-Aimé Rakotoarisoa, Chantal Radimilahy, Mark Stoneking, Thierry Letellier

**Affiliations:** 1 Laboratoire d’Anthropologie Moléculaire et Imagerie de Synthèse, UMR 5288 CNRS, Université de Toulouse, 31073 Toulouse, France; 2 Aix Marseille Univ., CNRS, EFS, ADES, Marseille, France; 3 Commissariat à l’Energie Atomique, Institut Génomique, Centre National de Génotypage, 91000 Evry, France; 4 Institut de Civilisations/Musée d’Art et d’Archéologie, Université d’Antananarivo, 101 Antananarivo, Madagascar; 5 Department of Evolutionary Genetics, Max Planck Institute for Evolutionary Anthropology, Deutscher Platz 6, D-04103 Leipzig, Germany

## Abstract

While admixed populations offer a unique opportunity to detect selection, the admixture in most of the studied populations occurred too recently to produce conclusive signals. By contrast, Malagasy populations originate from admixture between Asian and African populations that occurred ~27 generations ago, providing power to detect selection. We analyze local ancestry across the genomes of 700 Malagasy and identify a strong signal of recent positive selection, with an estimated selection coefficient >0.2. The selection is for African ancestry and affects 25% of chromosome 1, including the Duffy blood group gene. The null allele at this gene provides resistance to *Plasmodium vivax* malaria, and previous studies have suggested positive selection for this allele in the Malagasy population. This selection event also influences numerous other genes implicated in immunity, cardiovascular diseases, and asthma and decreases the Asian ancestry genome-wide by 10%, illustrating the role played by selection in recent human history.

## Abstract (web summary)

The population of Madagascar arose from admixture of Austronesian and Bantu genetic backgrounds. Analyzing local ancestry in genomes of 700 Malagasy, Pierron et al. identify signals of recent positive selection for African ancestry in a region on chromosome 1 with implications for physiology and disease risk.

## Introduction

Speaking an Austronesian language and living <400 km from the African coast, the origins and history of the Malagasy populations have long been a mystery. Recently, combined advances in archeology, genetics, and linguistics, along with extensive sampling across Malagasy populations, provide a better understanding of the settlement history^1–3^. Indeed, Malagasy populations originate from an admixture between populations with Austronesian and Bantu genetic backgrounds that occurred ~27 generations ago^4^. Linguistic and genetic evidence converge on a model of Bantu populations migrating across the Mozambique channel from the east coast of Africa, while the Austronesian population came 7000 km across the Indian Ocean from south Borneo^3^.

The meetings of these two populations led to both cultural and genetic mixture, generating a patchwork of cultural and genetic traits across Madagascar. For example, while the language is mostly Austronesian, the musical instruments are mostly from Africa^5^, and Austronesian genetic ancestry is higher in the central highlands than elsewhere^4^. The cultural and genomic landscape of Madagascar mostly reflects historic events of contact between groups followed by random fluctuations. However, some cultural aspects, such as farming, might reflect cultural adaptation to environmental constraints, as the adoption of crops and farming practices varied according to environmental circumstances^6^. Here we test the hypothesis that in addition to culture adaptation, genetic adaptation has also played a role in the history of Madagascar, by examining the distribution of Austronesian-related and Bantu-related genetic ancestry across Malagasy genomes.

In the absence of selection, the ancestry associated with each parental group of an admixed population is expected to be distributed more or less evenly across the genome; significant departures from this expected random distribution are thus a potential signal of selection. This approach has been used to demonstrate the importance of “adaptive introgression” of genes between modern populations^7–10^ or even between archaic humans and modern populations^11–13^. However, detecting admixture-related selection is problematic in modern populations where the admixture occurred only a few generations ago, as there may not have been enough time for selection to generate a detectable signal in the distribution of ancestry across the genome. For example, there is ongoing debate concerning results suggesting positive selection for African alleles at some loci in the Americas^7–9^.

As the admixture in Madagascar occurred ~27 generations ago^4^ and involves populations that diverged at least 50 000 years ago^14^, the admixture approach should be informative, as ancestry can be confidently assigned and there has been ample time for selection to generate a detectable signal. Indeed, signals of positive selection have been detected in the Malagasy populations^2, 15^. In particular Hodgson et al.^15^ showed that under neutrality the high frequency of the African Duffy null allele in the Malagasy population^16^ is incompatible with the admixture level measured in Madagascar; this allele is associated with protection against *Plasmodium vivax* malaria and is fixed (or nearly so) in Africa but essentially absent outside Africa^17^. This study analyzed variation at a single single-nucleotide polymorphism (SNP) in two Malagasy populations^15^, here, to further investigate this as well as other potential signals of recent positive selection, we performed a genome-wide analysis of local ancestry across the genomes of 700 Malagasy based on a sampling from 257 villages from all across Madagascar^4^. We find that the Malagasy genome has been shaped by a selection event, probably corresponding to selection for the Duffy null allele, that is exceptional in terms of strength (*s* > 0.2) and overall impact (~25% of the chromosome is in the selected region). Moreover, this selection has been ongoing over the last millennium, showing that recent selection does influence human populations.

## Results

### Local ancestry estimation

To look for signals of recent positive selection, we used the mixed ancestry of Malagasy genomes and searched for genomic regions with unusually high or low levels of African or Asian ancestry. To measure the Asian and African contribution across the Malagasy genome, we used the Efficient Local Ancestry Inference (ELAI) algorithm to perform local ancestry inference on 1.9 million SNPs genotyped in 700 individuals sampled from 257 villages across Madagascar^4^. The average Asian ancestry across individuals was 38.47 ± 3.0% per position across the genome, in agreement with previous studies^2, 4, 15^.

**Fig. 1:**
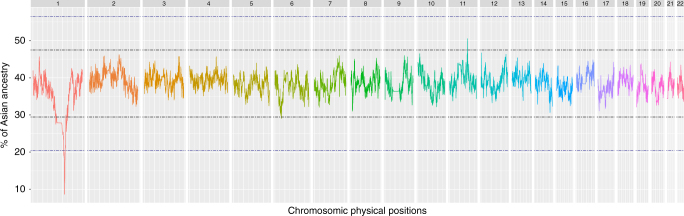
Average Asian ancestry estimated using ELAI, across the 700 Malagasy individuals, for each position of each chromosome. In this analysis the African and Asian populations from the 1000 Genomes Project^22^ were used as proxies for the African and Asian ancestry in the Malagasy; the results from other reference populations are provided in Supplementary Figure, and using other approaches and reference populations in Supplementary Figures 2 and 3. Black lines represent a deviation of 3 SD from the mean and blue lines represent a deviation of 6 SD from the mean

**Fig. 2:**
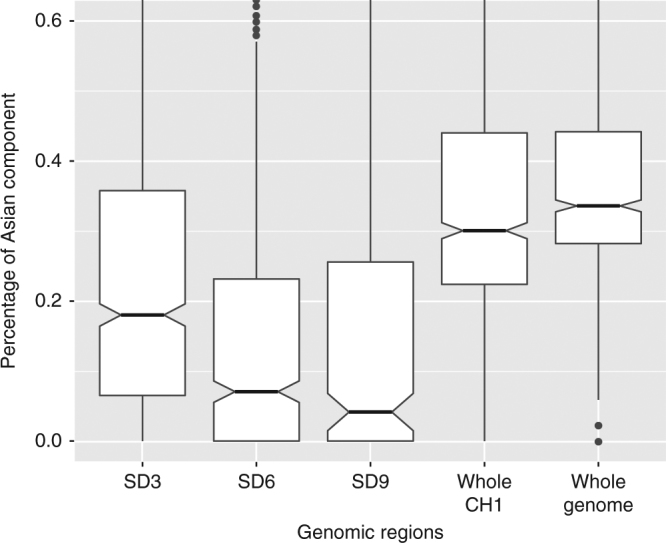
Asian ancestry proportion of the Malagasy population by genomic region, based on an unsupervised ADMIXTURE analysis for *k* = 2. SD3 (3 standard deviations from the average ancestry) represents the region chr1: 114423653–175653680; SD6 represents the region chr1: 154764878–161975281; and SD9 represents the region chr1: 158934324–160006961

The distribution of the ancestry inference across each position in the genome (Fig. 1) revealed a highly significant decrease in Asian ancestry (and corresponding increase in African ancestry) on chromosome 1 near the locus 1q23. The lowest Asian ancestry in this region is 8.7%, corresponding to a deviation of 9.9 standard deviations (SDs) from the mean and a *p*-value < 10^−16^ (Grubbs tests for one outlier *G* = 9.90, *U* = 0.99995 computed on the whole dataset and *G* = 10.00, *U* = 0.99971 on pruned dataset). A similar decrease in Asian ancestry was observed when different combinations of African and Asian populations were used as proxies (Supplementary Fig. 1), or different algorithms were used to infer local ancestry (Supplementary Fig. 2). This decrease in Asian ancestry was also observed when applying an unsupervised global ancestry method (ADMIXTURE, *k* = 2) on various windows over the genome (Fig. 2). Thus, the decrease in Asian ancestry near locus 1q23 in the Malagasy is robust to the choice of reference populations or local ancestry algorithm.

### Selection analysis

**Fig. 3:**
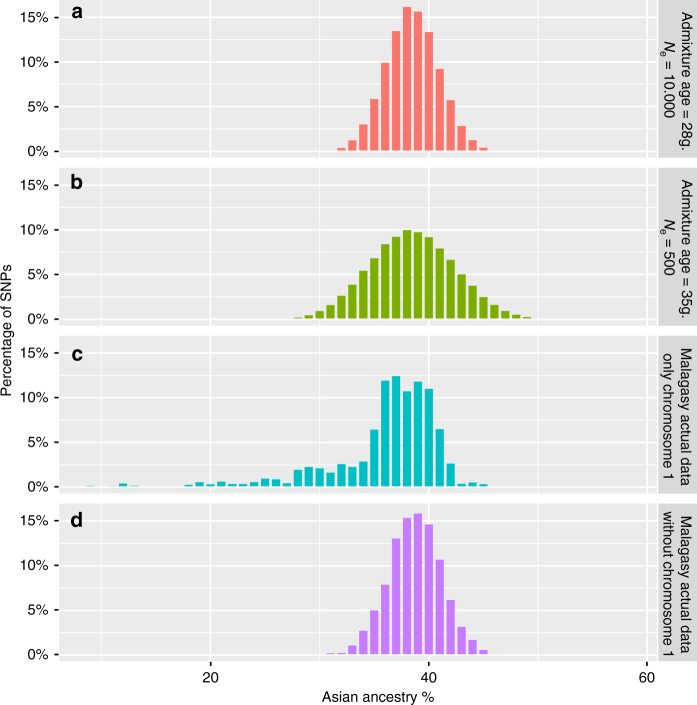
Distribution of Asian ancestry across the genome. **a** The simulated distribution based on the “realistic model”. **b** The simulated distribution based on the “drift model”. **c** The observed distribution for all positions on chromosome 1. **d** The observed distribution for all positions in the genome (excluding chromosome 1)

We next used computer simulations^15^ to estimate the probability that genetic drift alone could produce such a low frequency (8.7%) of Asian ancestry. We carried out one million simulations using a “realistic” model based on demographic parameters estimated from the same dataset. For this “realistic” model the date of admixture between Asian and African populations was based on previous estimations^4^ based on ALDER^18^ and GLOBETROTTER^19^ and represents the starting point of the simulations (27 generations ago). The history of population size change (average grow rate of 18% with an effective size of ~10 000 individuals) was inferred using non-parametric estimation (a refined IBD algorithm^20^). None of the simulations produced a final frequency of <20.6% Asian ancestry (Fig. 3). We then used demographic parameters that would increase the genetic drift by decreasing *N*_e_ and increasing the age of admixture (“drift model”:* N*_e_ = 500, admixture age = 35 generations, growth rate = 18%; note that while decreasing the growth rate would also increase the genetic drift, lower growth rates result in unrealistically low current population sizes), but again none of the one million simulations reached the extreme value observed in the actual Malagasy dataset. Moreover, the distribution of ancestry observed across Malagasy genomes is very close to that of the realistic model (Fig. 3), while in the drift model the SD is increased by 60% (actual data SD = 2.47%, realistic model SD = 2.43%, and drift model SD = 4.07%; Fig. 3). Thus, these results confirm that parameters used in the realistic model (in particular, recent admixture and large *N*_e_) produce simulated data more similar to the real data than the drift model, and exclude a significant role for genetic drift in the low Asian ancestry observed for the 1q23 region. Instead, it appears that selection has operated to increase African ancestry at the expense of Asian ancestry for this chromosomal region.

**Fig. 4:**
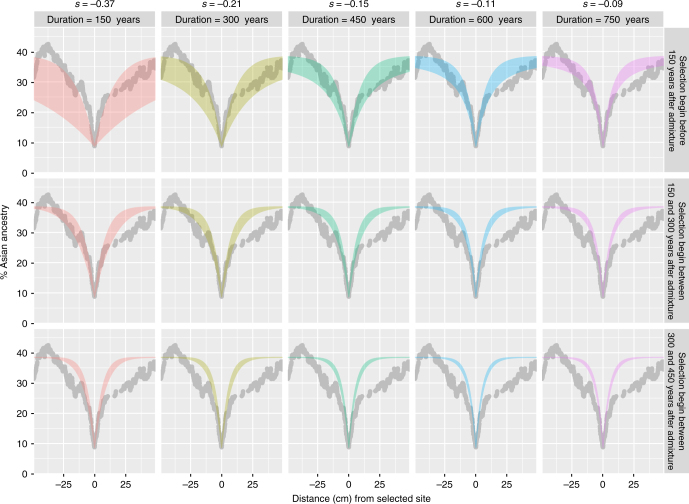
Change in local ancestry expected around a site under selection computed for various times of selection and selection coefficients. The gray dots represent the actual data for Asian ancestry observed around the position rs12705 (*ACKR1* gene), the colored bars are the expected values for the indicated selection coefficient, the interval between the onset of admixture and the onset of selection, and the duration of selection

Selection models^8^ indicate that extremely strong selection for African ancestry (*s* ≥ 0.09 for a dominant allele and *s* ≥ 0.625 for a recessive allele) is needed to explain how African ancestry can reach 92.3% in at most 27 generations since admixture. Moreover, selection might have been even stronger if it occurred over a shorter period of time. To explore this possibility, we compared the observed change of Asian ancestry to the expected change, as a function of different start times and selection periods (Fig. 4). The observed results are compatible with selection beginning at most 150 years after the admixture, as selection happening later would produce a sharper curve (Fig. 4). Overall, the results suggest strong selection (*s* > 0.2) happening immediately after admixture. Moreover, such strong selection at the 1q23 region during admixture would decrease not just the local but also the global Asian ancestry percentage during the first generations (Supplementary Fig. 3). That is, in the absence of selection, global Asian ancestry would likely be 5–10% higher than the currently observed 38% (Supplementary Figs. 3, 4), because in the initial generations individuals with high amounts of Asian ancestry across their entire genome were selected against. Thus, strong selection has likely influenced not just the local but also the global percentage of African/Asian ancestry in the present Malagasy population.

### Identifying candidate targets

**Fig. 5:**
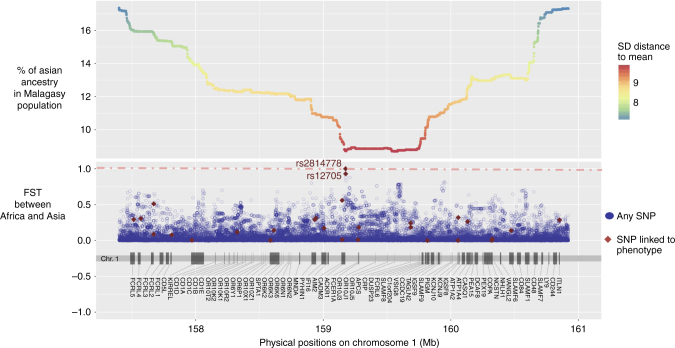
Distribution of the amount of Asian ancestry across Malagasy individuals for the 1q23 chromosomal region, and the Fst value between Asian and African populations for each SNP. SNPs marked in red have been linked to a phenotype by GWAS

This selection significantly reduced Asian ancestry (3 SDs from the mean, Fig. 1) over a large region of 61 Mb (chr1:114423653–175653680), encompassing ~25% of chromosome 1 and including the centromere as well as 1303 genes. In an attempt to further pinpoint the selected mutation, Isafe analysis^21^ was performed (supplementary Figure 4). This method exploits coalescent-based signals to rank all mutations within a 5 Mb around a region under selection and was designed to work in regimes where the selection strength is high. However, no significant signal was detected within the area exhibiting the highest African ancestry (>9 SDs from the mean), indeed all iSAFE scores obtain on this region (<0.32) are low scores below the 0.1 threshold. This lack of evidence might be due to the fact that the admixture process can hide the selective process particularly because the selection was for African haplotypes, which are more diversified than Asian haplotypes. We then searched the region with the highest African ancestry (>9 SDs from the mean) for SNPs exhibiting the highest differentiation between Asian and African populations in the 1000 Genomes Project^22^ as well as SNPs associated to any phenotype^23^ (Fig. 5). This step allowed us to pinpoint two SNPs: rs2814778 and rs12075, located in the *ACKR1* gene, that: (1) are the most differentiated between the Asian and African populations with Fst values of 1.00 and 0.93, respectively; (2) are also located in the peak of lowest Asian ancestry in Madagascar; and (3) are associated with a phenotype, namely the Duffy blood group-negative and A/B phenotypes, respectively. Genotyping the Malagasy population for these two SNPs indicates that the African alleles of both rs2814778 and rs12705 are at high frequency (91.4% and 93.4%, respectively); overall, we estimate that 85.6% of the Malagasy are Duffy-negative in agreement with previous results^16^. Individuals who are Duffy-negative lack the receptor used by the *P. vivax* malarial parasite to invade red blood cells, and hence are more resistant to this type of malaria^16^. In accordance, it has been already proposed that selection for such resistance is responsible for the high frequency of the Duffy-negative allele in Africa^24–26^ and Madagascar^15^, and the virtual absence of *P. vivax* across most of sub-Saharan Africa (but not Madagascar^16^).

### Spatial analyses

**Fig. 6:**
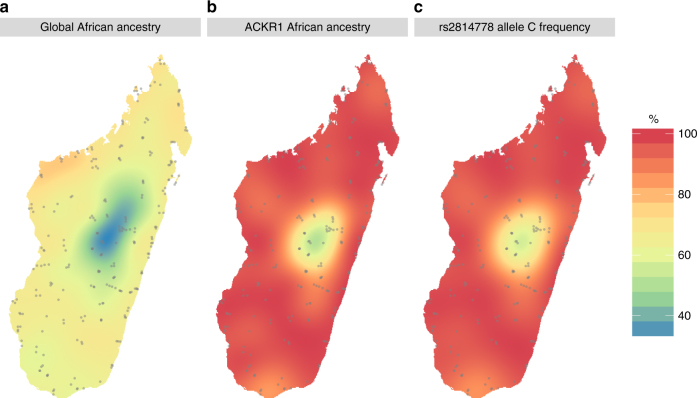
Spatial distribution of African ancestry percentage across Madagascar: **a** global ancestry computed on all positions (excluding chromosome 1); **b** local ancestry computed for the *ACKR1* locus; **c** frequency of the Duffy null allele. Sampled villages are represented by gray dots. The underlying map was generated by R using the library mapdata (2016)

We further analyzed the distribution across Madagascar of the decrease in Asian ancestry at the 1q23 locus by comparing the Asian/African ancestry estimation for the *ACKR1* gene to the global ancestry and to the Duffy null allele frequency. Previously, we showed that the Malagasy population is structured into at least 10 geographically distributed groups^4^. For all groups, the local African ancestry is higher for *ACKR1*, even for one group located in the highlands (g01), where the global Asian ancestry is dominant (Supplementary Table 1). To further investigate geographic distribution, we interpolated the African ancestry across Madagascar based on a Kriging model (Fig. 6). African ancestry for the *ACKR1* gene is higher than 95% for about 2/3 of Madagascar, and appears to be fixed (>99%) for ~10% of the country (Fig. 6). However, we caution that since reliable information on Madagascar population density is not available (the last census was in 1993 and the population has supposedly doubled since this date; INSTAT, Madagascar https://www.instat.mg), it is not possible to extrapolate the percentage of African ancestry across the landscape to actual population percentages.

Overall, local ancestry for the *ACKR1* gene as well as global ancestry are correlated with geography (Moran’s *I*, *p*-value < 10^−16^). The lowest African ancestry, both locally for the *ACKR1* gene and genome-wide, is in the highlands (Fig. 6). Nevertheless, while the African global ancestry is under 50% in the highlands, the estimated local African ancestry for the *ACKR1* gene and the Duffy null allele frequency are both over 50% (Fig. 6c). Overall, there is strong evidence of adaptive introgression of the African *ACKR1* gene all across Madagascar, even in regions with high Asian ancestry.

Because the central highlands show the highest Asian ancestry both at the 1q23 locus as well as genome-wide, it seems more likely that this higher Asian ancestry is linked to the settlement history of Madagascar and not to differences in selective pressures such as malaria. Indeed, we previously suggested that the population living presently in the highlands stems from a founding population with mostly Asian ancestry and only limited admixture from Africa^4^. Nevertheless, the desire to escape malaria might have driven the population movement to the highlands; presently, the coastal areas are most affected by malaria, and the Antananarivo region in the highlands is almost unaffected. Indeed, the two populations in the center (g01 and g02) exhibit the lowest intensity of selection (<0.1, Supplementary table 1). However, our current knowledge concerning the distribution of *P. vivax* malaria across Madagascar is limited and moreover historical reports suggest that the impact of malaria has changed during the last century due to urbanization as well as health and eradication campaigns^27^.

### Genes affected by selection

Although selection for the Duffy null allele seems the most likely explanation for the elevated African ancestry at the 1q23 locus, there are other genes that might have been involved in the selection and/or impacted by the selection. In addition to the Duffy blood group gene, the chromosomal region with the highest African ancestry (>90%, 9 SDs from the mean, Table 1) also contains disease-related biomarkers known to differ greatly between African and non-African populations: (1) C-reactive protein level (linked to the *CRP* gene), an inflammation marker that is higher in populations with African ancestry and associated with cardiovascular disease risk^28^; (2) immunoglobulin E levels, associated with *FCER1A* (Fc fragment of immunoglobulin E (IgE)) polymorphisms, are higher in populations with African ancestry and are associated with asthma susceptibility^29^; and (3) low white blood cell counts (benign ethnic neutropenia) are also linked to African ancestry at the 1q23 locus^30^. SNPs for all of these loci exhibit large frequency differences between East Asian and African populations, and the Malagasy exhibit allele frequencies more similar to those in Africans and significantly different from frequencies predicted by admixture without selection (Fig. 5 and Table 1). Moreover, African-associated alleles associated with at these four loci exhibit significant linkage disequilibrium (LD) with the Duffy null allele (Supplementary Table 2). This may explain the occurrence of phenotypes associated with elevated African ancestry, such as low leukocyte levels, even in highland populations^14^.

**Table 1:** Allele frequencies for SNPs present on the genotyping array that exhibit a strong association with a phenotype (Phen-gen *p*-value < 10^−8^), and are in the 1q23 chromosomal region

	SNP	Ref/alt	Associated phenotype	Observed frequency			Expected frequency (mean ± SD)	Probability of observed data
				Africa (%)	East Asia (%)	Madagascar (%)		
SD9	rs1101999	T/C	Asthma	31	0	32	19 ± 1.9%	<10^−6^
	rs3026968	C/T	Chemokine CCL2	3	53	8	22 ± 2.1%	<10^−6^
	rs3093059	A/G	C-reactive protein	32	15	35	25 ± 2.2%	2.10^−5^
	rs12075	A/G	Leukocyte count	2	92	7	37 ± 2.4%	<10^−6^
SD6	rs6684514	G/A	Erythrocyte indices	8	23	7	14 ± 1.7%	4 × 10^−6^
	rs1801274	G/A	Mucocutaneous lymph node syndrome	47	72	46	57 ± 2.5%	1.4 × 10^−5^
	rs7528684	G/A	Diabetes mellitus, type 1	6	56	11	25 ± 2.1%	<10^−6^
	rs1142287	T/C	Crohn disease	46	31	37	40 ± 2.5%	9.55 × 10^−2^
	rs12566888	T/G	Platelet aggregation	35	51	36	41 ± 2.5%	1.84 × 10^−2^
	rs13376333	C/T	Atrial fibrillation	30	3	23	20 ± 2.0%	4.87 × 10^−2^

The frequency observed in Madagascar is compared with that observed in Africa and East Asia, and to the expected frequency produced by admixture without selection (based on one million computer simulations^15^). Probability of observed data is based on the quantile of the observed frequency in the distribution of the simulation results

## Discussion

In this study, we show that chromosome 1 of the admixed Malagasy population exhibits an excess of African ancestry that cannot be explained by genetic drift alone, and instead represents a strong signal of recent positive selection (*s* > 0.2). The selection is for African ancestry and impacts at least one-quarter of the chromosome 1 (~60 million bp). Our analyses of local ancestry suggest that selection happened during the last millennium, beginning soon after the admixture between Asian and African populations. This result demonstrates that selective forces acting over <25 generations can drastically impact human genetic diversity. To our knowledge, this is one of the strongest signals of recent selection reported for modern humans.

The most probable candidate for this strong selection is the FY*O phenotype (Duffy null blood group), since the responsible SNP (rs2814778, in the *ACKR1* gene) is located in the peak of lowest Asian ancestry in Madagascar and is highly differentiated between Asian and African populations. Moreover, a previous study reported that a strong signal of positive selection influenced the Duffy null allele frequency in Madagascar^15^. Hodgson et al.^15^ observed that the frequency of the FY*O allele in Madagascar reported by epidemiological studies^16^ in two regions of Madagascar (Center and South) are higher than would be expected based on the global ancestry reported in these regions. Based on computer simulations, they showed that admixture followed by genetic drift could not explain the elevated frequency of the Duffy null allele in the Malagasy, and hence positive selection must be involved. Our analyses confirm their results, but we estimate a higher selection coefficient (*s* > 0.2 instead of the previous estimate of *s* = 0.066). Three factors likely contribute to this difference in estimated selection coefficient. First, the sampling of the original study was limited to two populations and compared FY*O allele frequencies to global ancestry estimates from other studies, and so may have been influenced by the genetic heterogeneity across Madagascar (Fig. 6). In contrast, we estimated both local ancestry and FY*O frequency using 700 individuals sampled over more than 250 villages across Madagascar. Second, Hogdson et al.^15^ used a starting time for the onset of selection of 33 generations ago, based on archeological evidence for the arrival of African cultural artifacts in Madagascar, whereas we used a starting time of 27 generations, based on actual estimates of when admixture started (using two different methods) from our same dataset. A shorter time for the same changes in allele frequencies will necessitate stronger selection (although this is unlikely to fully account for the discrepancy). Finally, Hogdson et al. studied one SNP, and thus could not estimate the size of the region influenced by selection; by contrast, our genome-wide analysis indicates that the selection impacted ~25% of the chromosome, about 60 megabases. All of these factors would lead to a stronger selection coefficient than was previously estimated. Overall, this is one of the strongest selective pressures estimated in human populations (for example: *s* = 0.09–0.19 for *LCT*; *s* = 0.02–0.05 for *G6PD* deficiency; *s* = 0.05–0.07 for the *MHC*; and *s* = 0.05–0.18 for the sickle-cell trait)^8, 31^.

As the African FY*O allele is associated with resistance to *P. vivax*, this provides a likely explanation for the strong selection. Long-term co-evolution between *P. vivax* and its receptor, the Duffy blood group, has been proposed to explain the distribution of *P. vivax* and the FY*O phenotype in Africa^13, 14^ and specifically in Madagascar^15, 16^. A recent study^17^ showed that the time to the most recent common ancestor (TMRCA) of human-specific *P. vivax* (70–250 kya) overlaps the TMRCA of FY*O (230 ± 61 kya), which is consistent with the supposition that malaria is responsible for the spreading of Duffy null blood group in Africa. The FY*O null allele then reached fixation in Africa during the last 50 000 years with a selection coefficient of 0.043 (95% confidence interval: 0.011–0.18)^17^, and led to the virtual elimination of *P. vivax* from the African continent due to the absence of the receptor needed to infect the host. While human *P. vivax* originated in African great apes^32^, *P. vivax* in the Malagasy likely originated from Asia^32–34^, and was probably carried by Indonesian settlers who were Duffy-positive^16^. African populations then brought the FY*O phenotype, which in the presence of *P. vivax* would have been strongly selected for in Madagascar as it was previously selected for in Africa^14^. An important health consequence of this scenario is that the several centuries of exposure of the mixed Duffy-negative and -positive Malagasy population to *P. vivax* may have enabled the parasite to overcome its dependence on the Duffy antigen, as *P. vivax* has been found in Duffy-negative individuals in Madagascar^16^. If this is indeed the case, the possibility exists that *P. vivax* could spread from Madagascar back to Africa.

Overall, this scenario is consistent with our results and could explain the strong signal of selection. However, questions remain concerning whether the prevalence of *P. vivax* and the associated mortality^35^ would be sufficient to have such a strong impact on the Malagasy genomes. Additional selective pressures may also be involved. For example, other *Plasmodium* (e.g *P. knowlesi*) also utilize the Duffy receptor to invade red blood cells^36^. Moreover, other phenotypes associated with genes in the 1q23 chromosomal region, such as those associated with CRP and IgE levels (Table 1), are also linked to parasite resistance in Africa^37^. In particular, the *FCER1A* gene encodes the Fc fragment of IgE and *FCER1A* polymorphisms are associated with IgE levels^38^; as IgE levels are associated with the response against a range of infections by parasitic worms (helminths), it is largely accepted that IgE levels have been under selective pressure to help counter metazoan parasites^37^ during human evolution. Although selection for the Duffy null allele seems most likely, a scenario with multiple forces contributing to the strong signal of selection for African ancestry in the 1q23 region should not be ruled out.

Regardless of the underlying cause(s), the selection for African ancestry at the 1q23 locus may have important consequences for the present physiology, health, and disease risks of the Malagasy population. The selected region contains several genes coding for disease-related biomarkers known to differ greatly between African and non-African populations (Table 1). Indeed, levels of CRP, IgE CCL2, and leukocytes are commonly used in modern medicine as biomarkers. In particular, high levels of IgE in African-Americans are linked to a high susceptibility to asthma^29^ and is believed to be a side product of adaptive selection against parasitic worms that happened in Africa before the transoceanic slave trade^37, 39^. Similarly, African ancestry at the *ACKR1/FCER1A* locus has been linked to low white blood cell counts in healthy African-Americans (benign ethnic neutropenia)^30^. In accordance with this idea, leukocyte levels reported in Madagascar are close to those reported for African populations^40^. Because white blood cell count is a common biomarker of infection, immunocompetence, and inflammation, the knowledge of this condition is an important part of the medical decision-making process in the United States^30^. In consequence, the elevated African ancestry at 1q23 should be considered when medical decisions are based on biomarkers such as CRP, leukocyte, or IgE levels, even for highland populations with mainly Asian ancestry.

Moreover, this selection in favor of African ancestry decreased the Asian ancestry not just at the 1q23 locus, but also genome-wide. Indeed if selection had not occurred, Asian ancestry genome-wide in Madagascar would probably be 45–50%, as opposed to the observed 38%. This is because in the initial stages of selection, individuals with Asian ancestry at the 1q23 locus that were selected against also had high levels of Asian ancestry genome-wide. In other words, African populations coming to Madagascar might have had a significant advantage over Asian people. It is not clear whether African or Asian populations first settled Madagascar, but it is possible that when the two populations came into contact in Madagascar, fitness differences played a role in the history of the settlement of Madagascar even before the admixture. Biology can thus play a role in historical events and should be considered when discussing the settlement of a new environment such as Madagascar.

In conclusion, in the present study we take advantage of the admixed history of populations from Madagascar to identify a strong adaptive selection that happened during the last millennium and impacted the entire country (some 25 million individuals). This illustrates how selection can impact the human genome; in particular, selection should not be seen only as a slow process acting over a large number of generations, but instead as a force which can sometimes act rapidly even during very recent human history.

## Methods

### Samples and generation of genome-wide data

The 700 samples analyzed here were collected from 2007 to 2014 utilizing an extensive grid-based sampling approach, in which individuals were sampled from 257 villages (2.8 ± 0.7 individuals per village) from all around Madagascar^4^. All individuals were unrelated and given detailed information on the study, and all gave written consent prior to donating their sample. This study was approved by the Human Subjects’ Ethics Committee of the Health Ministry of Madagascar as well as by French Ethics Committees (Committees of Protection of Persons, and French National Commission on Informatics and Liberty). Genome-wide SNP data were generated using the Illumina Human Omni 2.5–8 (Omni 2.5) BeadChip array; analyses were performed using PLINK 1.9^41^. All genotyped individuals and SNPs passed standard quality filters^4^. The dataset is available from EGA (EGAS00001002549). While rs12705 genotypes (A/B Duffy polymorphism) are directly available on the Illumina Human Omni array, the rs2814778 (Duffy null polymorphism) was independently genotyped on the same Malagasy individuals (with sufficient DNA), i.e., 681 individuals. We used a TaqMan SNP predesigned genotyping assay following the manufacturer’s protocol (Life Technologies, Cat #4351376) on the ABI Prism 7900HT platform.

### Comparative data

The Malagasy data were included in two separate datasets with putative source populations for local ancestry analysis. Both datasets included 24 Mandarese from Sulawesi Island in Indonesia analyzed with the same Illumina Human Omni 2.5-8 array as the Malagasy^4^. The first dataset (1 912 812 SNPs) is based on the 1000 Genomes Project^22^ with 30 individuals from each population (Africa: Esan from Nigeria, Yoruba from Nigeria, Gambian, Luhya from Kenya; Asia: Mandarese from Indonesia, Kinh from Vietnam, Dai from China, Han from China). The second dataset (184 658 SNPs) is from a previous study^4^ and includes Southeast Asian populations and a more diverse set of eastern African populations (Asia: Mandar, Chinese, Igorot, South Kalimatan Dayak, and Malays; Africa: Kenyan Bantu, South African Bantu, San, and Somali).

### Estimating local ancestry

Local ancestry assignment across the genome was performed using various algorithms. We first used the ELAI algorithm^42^ and unphased data from the 1000 Genomes Project^22^, with a pool of all African individuals as the African source and a pool of all Asian individuals as the Asian source. A second analysis was performed using ELAI and individual populations for each source population: (a) Esan vs. Chinese from Bejing; (b) Esan vs. Mandar; (c) Luhya vs. Mandar; and (d) Yoruba vs. Mandar. A third analysis was performed using the same pool of individuals as in the first analysis, but using RFMIX^43^ instead of ELAI to assign the local ancestry; the data were first phased with Beagle version 4.1^44^. RFMix was run twice, first with the PopPhased option selected in the command, and then with the TrioPhased option with five iterations. For both analyses other flags were as follows: --generations 25, --window-size 0.01; --min-node-size 5. Finally, the second dataset (with a focus on Southeast Asia and East African source populations) was analyzed; the data were phased with ShapeIT^45^ and PCADMIX^46^ was used to evaluate local ancestry.

Furthermore, as an independent test, we used an unsupervised global ancestry method to perform the estimation and the magnitude of deviations across the genome. We performed an unsupervised ADMIXTURE^47^ analysis for *k* = 2 using the same pool of individuals as in the first ELAI analysis (1000 Genomes pool) for various subsets of the data: -the entire genome; only on chromosome 1; chr1: 114423653–175653680 (the region deviating from the genome-wide ancestry estimate by at least 3 SDs: SD3); chr1: 154764878–161975281 (SD6 region); and chr1: 158934324–160006961 (SD9 region). Finally, Isafe analysis was performed on the 5 Mb region around the SD9 region from 156970642 to 161970642 and using ancestral alleles from ensemble database GRCh37^21^.

### Statistical analysis and modeling of local ancestry

Statistical analyses (such as mean and SD of local ancestry across the genome) were performed using R and plotted using ggplot. The presence of outliers was tested using the Grubbs tests from the outlier R package^48^. Mean, SD, and outlier statistics were computed on all SNPs present in the dataset (1 912 812) as well as a subset of 349 905 SNPs after trimming for LD by plink (--indep-pairwise 50 10 0.1). The expected distribution of local ancestry in a model of admixture without selection was obtained using the algorithm from Hodgson et al.^15^. Such models of the distribution of local ancestry require demographic parameters such as the following: (1) starting point of admixture; (2) population grow rate; and (3) initial effective population size. We ran a realistic model based on estimates from a previous study^4^ of the same dataset. The starting point of the simulations represents, by definition, the admixture between Asian and African populations. Based on ALDER^18^ and GLOBETROTTER^19^ analyses the date of admixture in Madagascar was estimated to be 22–27 generations ago. The history of population size changes in the Malagasy population was inferred using non-parametric estimation based on a refined IBD algorithm^20^. Demographic estimation showed an average grow rate of 18% with an effective size of ~10 000 individuals starting 27 generations ago. Based on these estimates we ran two models: (1) a “realistic” model based on parameters for the Malagasy population estimated previously^4^ (admixture 27 generations ago; initial effective size 10 000; growth rate 18%); (2) a “drift” model where parameters were exaggerated to increase the possibility of drift and thus the existence of outlier values (admixture 35 generations ago; initial effective size 500; growth rate 18%). The results were compared to the actual data from chromosome 1 and also from the rest of the genome using data trimmed by plink (--indep-pairwise 50 10 0.1).

### Genomic and phenotype analyses

The list of genes, phenotypes, and SNPs associated with phenotypes localized across the genome was extracted from the PheGenI database^23^. Fst values between Asian and African populations were computed using the VCFtools software^49^ and a pool of Luhya and Yoruba to represent Africa and a pool of Kinh and Han to represent Asia. Based on African and Asian allele frequencies as well as the admixture proportion (38.47% of Asian ancestry), we computed an expected frequency for the SNPs associated with a phenotype by genome-wide association study, present on the Illumina Human Omni 2.5 array, and located in the SD6 and SD9 regions of 1q23. Frequencies were computed using the algorithm from Hodgson et al.^15^ with one million simulations and the “realistic” model parameters (admixture 27 generations ago; initial effective size 10 000; growth rate 18%).

### Spatial analyses

For each individual, we estimated their global ancestry by averaging their local ancestry across the genome (excluding positions on chromosome 1). Estimation of local ancestry for the *ACKR1* gene was based on the local ancestry at the rs12705 locus (which is present on the Illumina Human Omni array). These statistics were interpolated across Madagascar using the exponential Kriging model in the package geoR and were also computed for each population defined previously^4^. Interpolation was used to compute the percentage of the landmass presenting an African ancestry over 95 and 99% (Fig. 6c). Based on the interpolation, the African introgression area in Fig. 6c was defined as the area where African local ancestry is over 50% while African global ancestry is <50%.

### Modeling the change in local ancestry

We first computed the selection coefficient necessary to explain the observed decrease in Asian ancestry on one locus under selection, assuming different time periods for the duration of selection. We followed previous methodology^8^ for a dominant mutation, (i.e., heterozygotes and homozygotes for the advantageous allele have the same fitness, 1 + *s*).

The expected change in local ancestry was then computed by modeling, generation after generation, the effect of the estimated selection on positions located at different distances from the selected site. This effect is directly proportional to the recombination rate (genetic distance expressed in Morgans) between a studied locus and the selected locus. Indeed, the genetic distance between two chromosome positions corresponds to the expected average number of intervening crossovers each generation. In consequence for each generation, the probability of a position to segregate along with the selected site corresponds to the distance between them in centimorgans. Computation steps are described in Supplementary Figure 5 and the code is provided (Supplementary Note 1). This expected change in African ancestry over time was then compared to the observed change in Malagasy populations.

### Data availability

The dataset is available from EGA (EGAS00001002549).

## Electronic supplementary material

Supplementary Information
