# Supplementary material for: Strong selection during the last millennium for African ancestry in the admixed population of Madagascar
Source: Nat Commun. 2018 Mar 2;9:932. doi: 10.1038/s41467-018-03342-5 (PMC5834599; doi:10.1038/s41467-018-03342-5)
Supplement: Supplementary file 1 — Supplementary Information [file 41467_2018_3342_MOESM1_ESM.pdf]

Strong selection during the last millennium for African ancestry in the admixed population of Madagascar

Pierron et al.

Supplementary Figure 1: Asian ancestry (%) across the genome using the ELAI algorithm and diverse source populations: a) Esan in Nigeria & Chinese from Bejing; b) Esan & Mandar from Sulawesi; c) Luhya in Webuye, Kenya & Mandar; d) Yoruba in Ibadan, Nigeria & Mandar. Black lines represent a deviation of 3 SD from the mean and blue lines represent a a deviation of 6 SD from the mean.

a) ESN & CHB

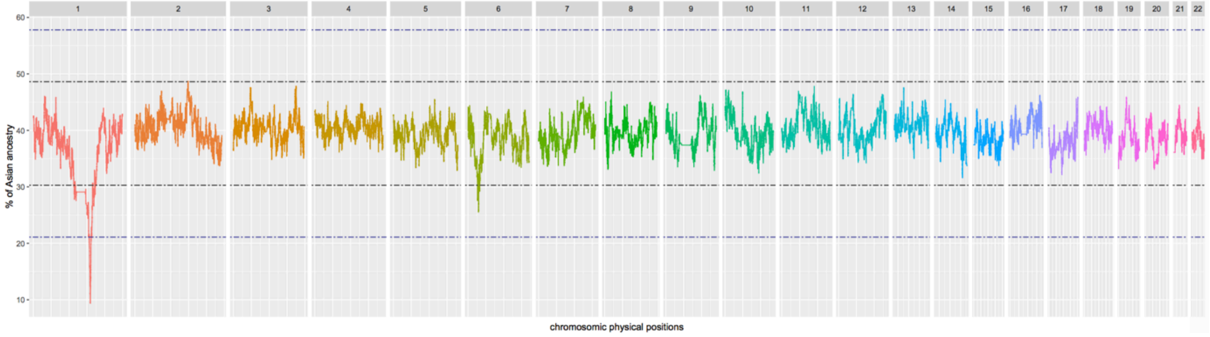

b) ESN & MANDAR

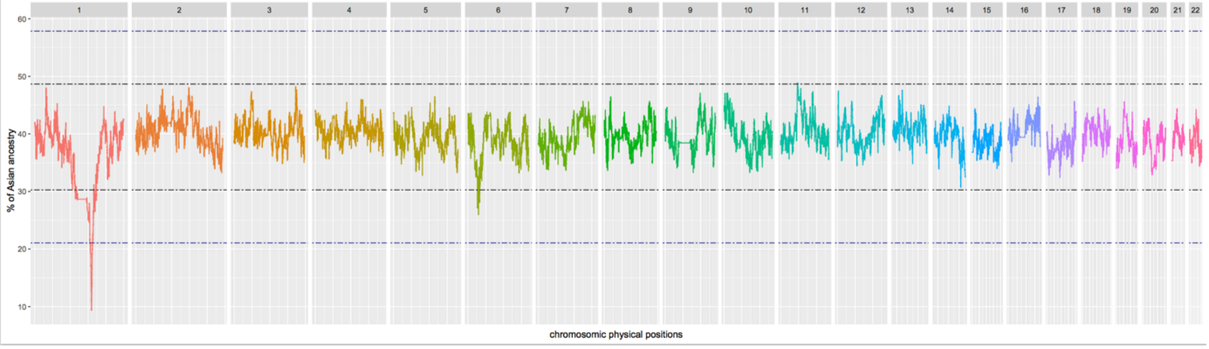

c) LWK & MANDAR

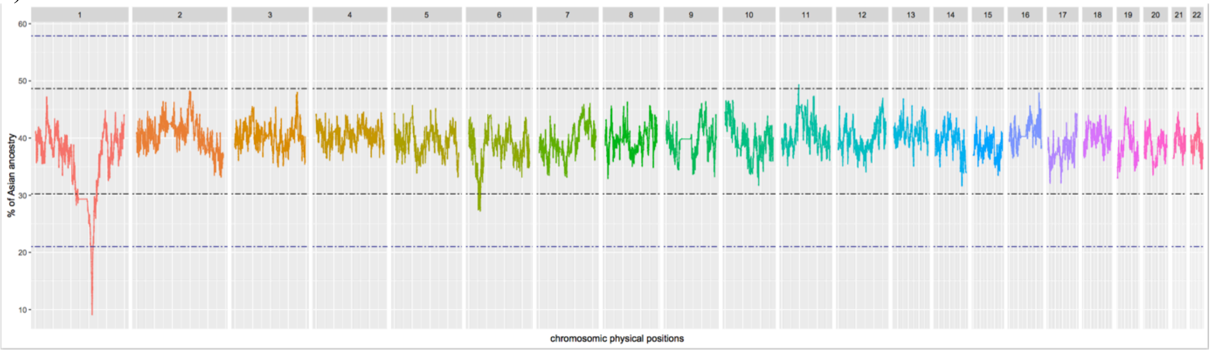

d) YRI & MANDAR

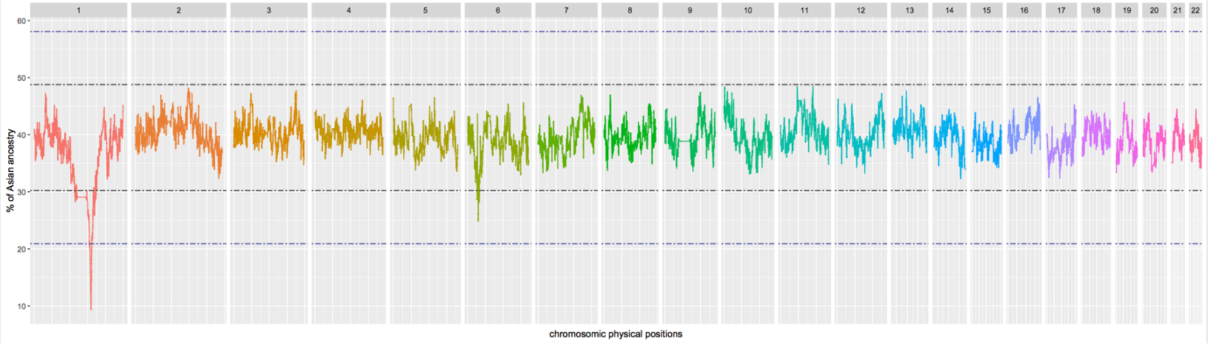

Supplementary Figure 2a: Asian ancestry (%) across the genome using PCAdmix and BEAGLE.

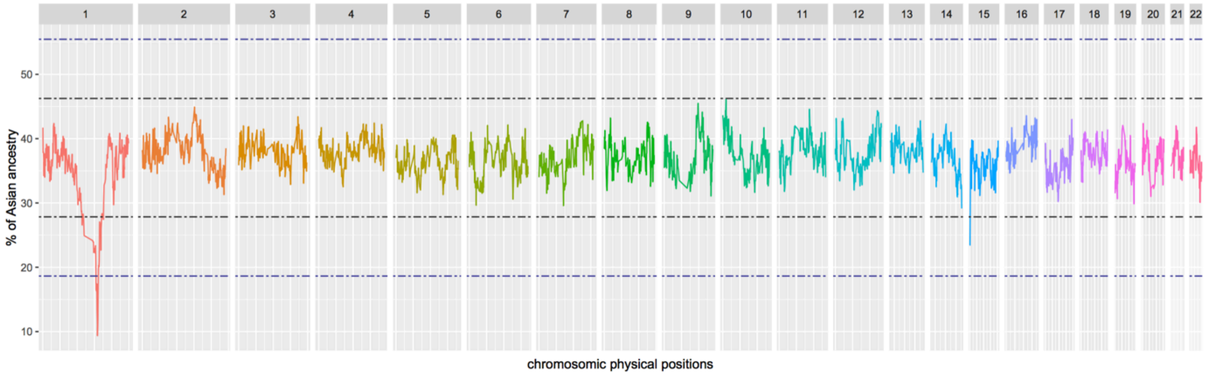

Supplementary Figure 2b: Asian ancestry (%) across the genome using RFMIX (POPphased option), SHAPEIT, and a different panel of source populations.

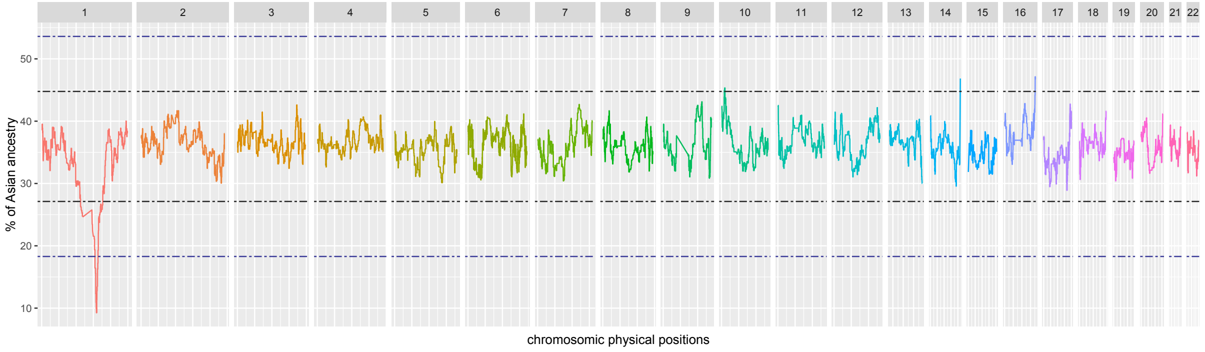

Supplementary Figure 2c: Asian ancestry (%) across the genome using RFMIX (TRIOphased option and 5 iterations), SHAPEIT, and a different panel of source populations.

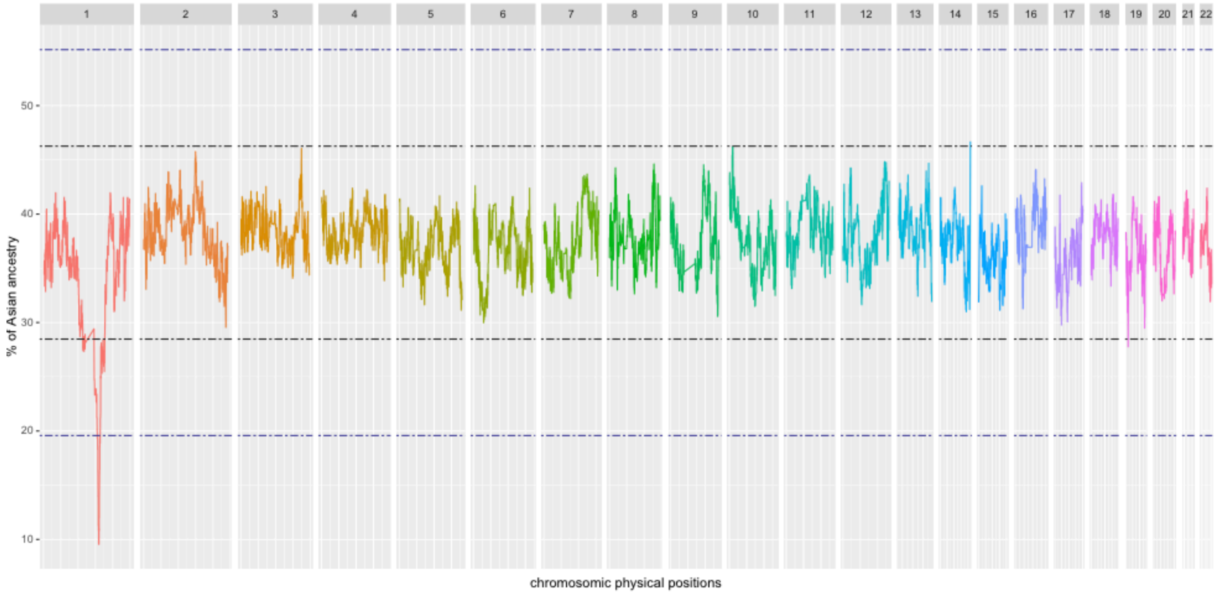

Supplementary Figure 3: Decrease of Asian ancestry over time after admixture due to the existence of one site under selection during different time periods (5, 10, 15, 20 or 25 generations). The blue rectangles represent the time during which selection operates for each scenario. Purple line: the Asian ancestry for the site under selection. Orange line: the average Asian ancestry at unlinked positions on other chromosomes.

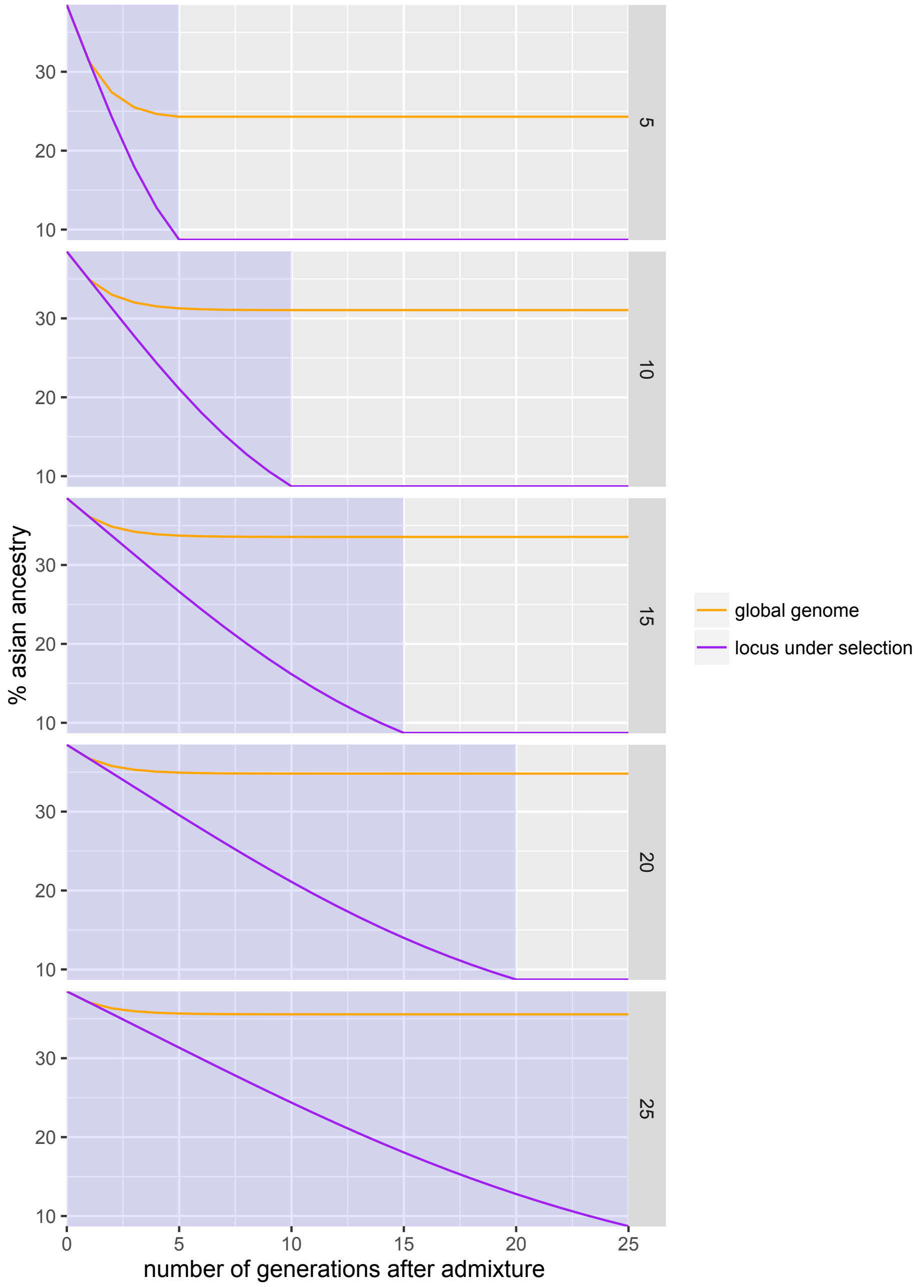

***Supplementary Figure 4*:** results of the Isafe analysis

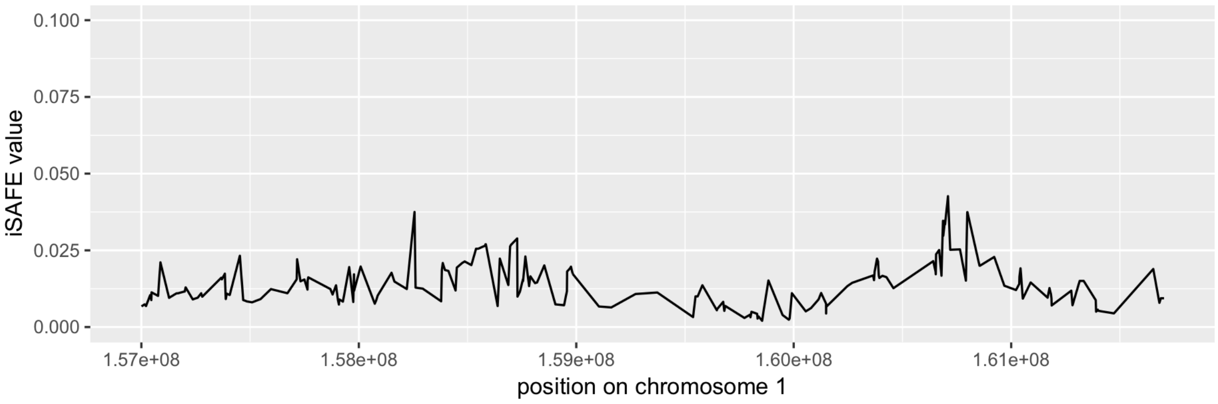

***Supplementary Figure 5*:** Outline of the procedure used to compute the expected change in Asian ancestry over time for a position at a specified distance from the selected site.

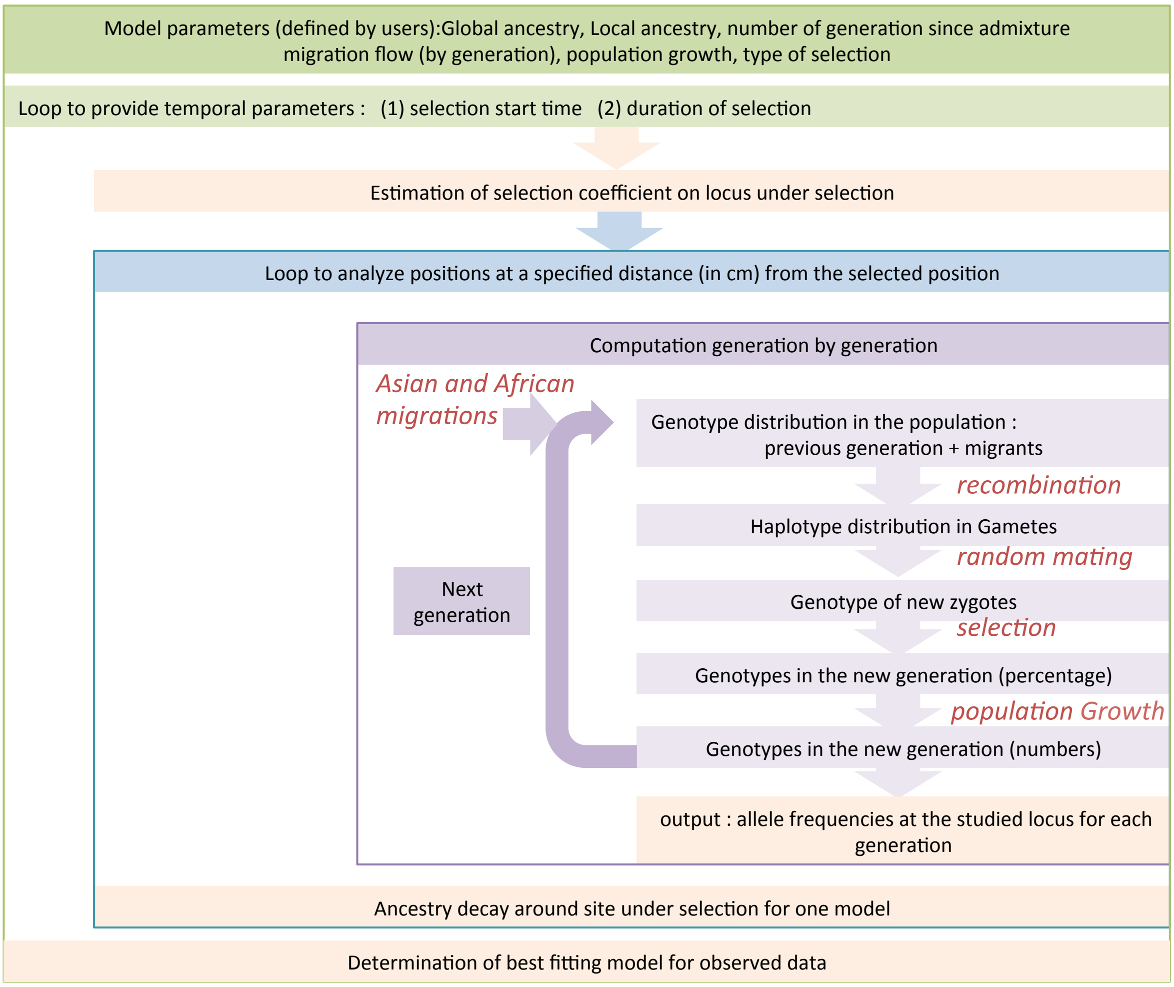

Supplementary Table 1: Analyses based on 10 Malagasy groups defined by previous fineSTRUCTURE and GLOBETROTTER analyses[1,2]. The time of admixture and estimates of Asian ancestry are from the previous GLOBETROTTER analyses. Also shown are estimates of Asian ancestry from ELAI for the entire genome (excluding chromosome 1), the ACKR1 gene, and the rs2814778 SNP, and estimated selective forces for both recessive and dominant models of selection against Asian ancestry.

		**GLOBE TROTTER**		**% of Asian ancestry (ELAI)**			**selective value**	
**pop.**	**n of indviduals**	**Time (gen.)**	**% asian ancestry**	**genome-wide**	**ACKR 1**	**Freq. rs2814778**	**Recessive model**	**Dominant model**
g01	67	23.90	64 %	68.2 %	50.7 %	49.2%(n=63)	-0.076	-0.05
g02	77	24.53	47 %	48.6 %	18.8 %	18.8%(n=77)	-0.084	-0.18
g05	61	24.79	40 %	38.9 %	9.0 %	10.2%(n=59)	-0.095	-0.41
g09	71	25.28	40 %	39.1 %	4.9 %	3.6%(n=70)	-0.117	-0.74
g06	86	23.85	33 %	30.6 %	2.9 %	2.9%(n=85)	-0.126	-1.00
g10	64	26.80	37 %	35.8 %	0.8 %	0.8%(n=61)	-0.169	-1.00
g03	92	22.20	28 %	25.4 %	0.5 %	1.1%(n=90)	-0.183	-1.00
g04	45	26.76	37 %	36.1 %	0.0 %	0.0%(n=40)	-0.313	-1.00
g08	81	25.68	35 %	33.5 %	0.0 %	0.6%(n=79)	-0.348	-1.00
g07	56	25.40	35 %	33.4 %	0.0 %	0.0%(n=56)	-0.367	-1.00

Supplementary Table 2: Plink estimation of Linkage desiquilibrium (D and r^2) between the rs2814778 Duffy null SNP and nearby SNPs associated with phenotypes. r^2 is limited due to the frequency of rs2814778.

**SNP**	**r^2**	**D**
rs6684514	0.000161352	0.150272
rs7528684	0.163605	0.442445
rs1101999	0.03007	0.820309
rs3026968	0.489539	0.752895
rs12075	0.694225	0.974932
rs3093059	0.0112769	0.46378
rs1801274	0.026796	0.499147

Supplementary Note

Supplementary Note 1 : Python code used to compute the expected change in Asian ancestry.

```
# -*- coding: utf-8 -*-
## this code is a suplementary file for the article entitled
#"Strong selection for African ancestry on chromosome 1 in the admixed population of Madagascar"


# Contributor(s):
#   Denis Pierron, Thierry Letellier
#   Laboratoire d´Anthropobiologie Moléculaire et Imagerie de Synthèse
#   CNRS UMR 5288,  Université Toulouse III
#   37 allées Jules Guesdes,  31073 Toulouse, France
#   Email : denis.pierron@cnrs.fr


# this code allow to compute the selection coeficient needed to switch the frequency of the ancestry of a SNPs
# it allows also to see it effect on surrounding positions

#################################################################
"PARAMETERS TO FILL"

# percentage of Asian individual arriving in Madagascar
# correspond to the global ancestry estimated
perASIE    = 0.3847017480125
# local ancestry percentage target in selection
# minimum of local ancestry on the locus under selection
perOBJ     =  0.0871078571428571

# number of generations to compute
# generally correspond to age of admixture
generations  = 27

# individual flows at every generation
# if fluxASIE =[1] this mean that all individuals came in only one generation
# and the mix is panmictic then
# if fluxASIE =[1,2,2,1] allow to spread the admixture on 4 generations with more individual coming in the middle
fluxASIE    = [1]  #[1] #
fluxAFRIQUE = [1]  #[1,1,1,1,1,1,1,1] # #


# type of selection
typeSELECTION = "dominant"
# work also with typeSELECTION ="recessif"


# name that you want give to file output
model = "MAGE"
# folder where you want the OUTPUT
```

```
dossier = "/Users/denis/"



###############################
"--- stop ---"
"there is no more parameter to file after this line"
"you sould not change anything unless you know what you are doing"


###############################
print "simulation during ",generations,"generations of one",typeSELECTION,"mutations"
###############################



# computing for a set of specific oparameters

def calcul(startSELECTION,stopSELECTION,recombinaison,selection):

        "------------------------------------------------"
        "A)) definitions of SNPs to be studied"
        "------------------------------------------------"

        "A.1) the genotype of each source population"
        asie= "AC_AC"; afrique = "BD_BD"

        "A.2) the one SNP under selection"
        # 2 possible alleles
        # A is from Asia and B is from Africa
        SNP_1 = ["A" ,"B"]

        # allele A is the reference (dominant / recessive) and is from Asia
        # careful selection of the coefficient might be negative (if negative selection)
        if typeSELECTION ==  "dominant":
           # homozygote and heterozygote share same fitness
           sel= {"AA": 1 + selection, "BB": 1 ,
              "AB": 1 + selection, "BA": 1 + selection}

        elif typeSELECTION ==  "recessif":
           # only homozygote are under selection
           sel= {"AA": 1 + selection, "BB": 1 ,
                 "AB": 1 , "BA": 1 }


        "A.3) the other SNP, which is not under selection"
        # 2 possible alleles
        SNP_2 = ["C","D"]


        "A.4) All possible combinations of haplotypes and genotypes"
        # List of haplotypes that is to say SNP on the same chromosome
        # here haplotype is composed of two SNPs
        # actually haplotypes = ["AC","AD","BC","BD"]
```

```
haplotypes = [ X+Y for X in SNP_1 for Y in SNP_2 ]

# lists of genotype that is just the combination of an individual's two chromosomes
# actually genotypes = ['AC_AC', 'AC_AD', 'AC_BC', 'AC_BD', etc... '
genotypes  = [ h1+"_"+h2 for h1 in haplotypes for h2 in haplotypes]


"A.5) fitness of each possible genotype "
fitness   ={}
for genoT in genotypes:
        # the selection coeficient genotype actually on depend o first SNP (position 0 and 3 of
genoT)
        # if genoT = 'AC_AD' then genoS = "AA"
        genoS = "".join([genoT[0],genoT[3]])
        # attribution of fitness value
        fitness [genoT] = sel[genoS]

"A.6) number of individuals for each genotype"
# number of genotypes at the start
# actually this is 0, because first individuals came with first migration
effectifs0 ={}
for genoT in genotypes:
    effectifs0 [genoT] = 0

"-----------------------------------------------"
"B)) PROBABILITY OF PRODUCTION OF EACH TYPE GAMETES"
"-----------------------------------------------"

# this step prepar a dictionary which defines the different gametes that a person can produce
according to its own genotype
# it takes into account the probability of recombination between the 2 SNPs

# proportion of gamete from each genotype
PROPgamet ={}

for genoT in genotypes:
    PROPgamet [genoT] = {haploT:0  for haploT in haplotypes}

    "B.1) list of haplotypes possible without any recombination"
    # there is always only 2
    #if genoT = 'AC_BD' then h1= AC and h2 = BD
    h1 = genoT[0]+genoT[1]  ; h2 = genoT[3]+genoT[4]
    # probability of gamete production
    # which is the probability of no visible recombination betwen the site
    # we divide by 2 because h1 an h2 are equiprobable
    PROPgamet [genoT][h1] += (1 - recombinaison/float(100))/2
    PROPgamet [genoT][h2] += (1 - recombinaison/float(100))/2

    "B.2) list of haplotypes possible with recombination"
    # there is always only 2
    #si genoT = 'AC_BD' alors h3= AD et h4 = BC
    h3 = genoT[0]+genoT[4]  ; h4 = genoT[3]+genoT[1]
    # probability of gamete production
    # we divide by 2 because h3 an h3 are equiprobable
    PROPgamet [genoT][h3] += (0 + recombinaison/float(100))/2
```

```
    PROPgamet [genoT][h4] += (0 + recombinaison/float(100))/2


"----------------------------------------------"
"C)) COMPUTE MIGRATIONS AND FLUX "
"----------------------------------------------"

"C.1) effective population size coming from each source population"
Nasie=Ninitial*perASIE   ;     Nafrique= Ninitial -Nasie

"C.2) number of individuals that the user has actually enter"
# we consider that the user puts proportions
# [1,2,0.5] means: 1 packet/batch arrives first and after twice and after half
# in the end 3.5 people packet came to Madagascar from asia
Sasie    =float(sum(fluxASIE))
Safrique=float(sum(fluxAFRIQUE))

# but the 3.5 packet actually mean thousands of individual X
# so you have to calculate the number of individual by packet
# so we calculate a ratio to convert that in total
RatioAsie= Nasie /Sasie
RatioAfrique=Nafrique / Safrique

"C.3) list of individuals who come generation after generation"
Ef_asie=[]
Ef_afrique=[]
# we use try/except since the user probably does not meet all the generations with migration
for gener in range(generations+1):
    # MIGRATIONS FROM ASIA
    try:
        # number of packs multiply by the number of individuals in one pack
        Ef_asie += [ fluxASIE [gener] * RatioAsie ]
    except:
        # if the box does not exist then no migration = 0
        Ef_asie += [0]

    # MIGRATIONS FROM AFRICA
    try:
        #number of packs multiply by the number of individuals in one pack
        Ef_afrique += [fluxAFRIQUE [gener]* RatioAfrique ]
    except:
        # if the box does not exist then no migration = 0
        Ef_afrique += [0]

"----------------------------------------------"
"D)) ADMIXURE AND SELECTION BY GENERATION "
"----------------------------------------------"
retour     = ""  # <- table of output

"D.1 parameter of generation 0 "
# number of individual for each genotype
ados       = effectifs0
# effective size of initial population
taillePOP1 = 0
```

```
    ligne= [recombinaison,selection,startSELECTION,stopSELECTION-
startSELECTION,0,taillePOP1,round(perASIE,5), round(perASIE,5)]
    l  = [str(x) for x in ligne]
    #print l
    retour += "\t".join(l)+"\n"

    "D.2 generation by generations "
    for gener in range(generations):

        "D.2a parameters of the initial generations (teenagers(ADOS) of the previous)"
        effectif  = ados
        taillePOP = taillePOP1

        "D.2b the new migrant come"
        # migration flux
        africains = Ef_afrique[gener]  ; asiatiques = Ef_asie [gener]
        # computation of the new population size
        taillePOP = taillePOP + africains + asiatiques
        # add to genotype the "source" african (all with the same genotype)
        effectif [afrique] += africains
        # add to genotype the "source" Asian (all with the same genotype)
        effectif [asie] += asiatiques


        "D.2c GAMETES calculations of this new population"
        gametes = {}
        for gamT in haplotypes :
            # number of producted gametes
            N = 0
            for genoT in genotypes :
                N += effectif [genoT] * PROPgamet[genoT][gamT]
            # proportion to the entire population
            gametes [gamT]= N /float(taillePOP)
            #print gamT,N,gametes [gamT]

        "D.2d proportions of zygotes from gamete"
        # before selection
        # random mix
        zygotes={}
        for spermatozoides in gametes :
            for ovules  in gametes :
                oeuf = spermatozoides+"_"+ovules
                zygotes [oeuf] =  gametes [spermatozoides]  * gametes [ovules]
                #print oeuf,zygotes [oeuf]


        "D.2f zygotes selection: number of survivors CHILDREN"
        enfants={}
        taille_naturelle=0
        for genoT in genotypes:
            if gener >= startSELECTION and gener < stopSELECTION :
                #print "selection"
                # SELECTION in action
                enfants [genoT] = zygotes [genoT] * fitness [genoT]
            else:
```

```
            # no SELECTION
            enfants [genoT] = zygotes [genoT]
          taille_naturelle += enfants [genoT]

        "D.2g TEENS (ADOS): size adjustment by contributing to the expected growth"
        #lets keep in growth expected sizes Despite the selection
        # But it does not influence the expected percentage
        taille_souhaite =  taillePOP * croissance
        ratio = croissance / taille_naturelle
        taillePOP1 = 0;  ados = {}
        for genoT in genotypes:
          T = enfants [genoT] * ratio * taille_souhaite
          taillePOP1  += T
          ados [genoT] = T


        "D.2h calculating allelic frequency in this generation"
        allele1 = SNP_1 [0] ;per1 =0
        allele2 = SNP_2 [0] ;per2 =0
        for genoT in genotypes :
            per1 += ados [genoT] * genoT.count(allele1) / float(taillePOP1 * 2)
            per2 += ados [genoT] * genoT.count(allele2) / float(taillePOP1 * 2)

        "D.2i => saving generation results"
        ligne= [recombinaison,selection,startSELECTION,stopSELECTION-
startSELECTION,gener+1,taillePOP,round(per1,5), round(per2,5)]
        l  = [str(x) for x in ligne]
        retour += "\t".join(l)+"\n"


    return(per1,retour)


#####################################################
##LAUNCH BY AMENDING THE TIME SELECTIONS
#####################################################
# original effective population size (does not influence anything yet)
Ninitial = 10000  ; croissance = 1


"AA) output to final file "
tout=""

"BB) the different possible selection time spans"
for startSELECTION in range (0,20,5):
    dureeLIST = range (5,generations+4,5) + [generations+1]
    for dureeSELECTION in dureeLIST:
        stopSELECTION = startSELECTION+dureeSELECTION

        "---------------------------------------------"
        "BB) finding the right selection coeficient for the expected result "
        # changing the correct coeficient using the zhou et al. 2016 method
        SELECTIONmin = 1  ;  SELECTIONmax = -1
        for xx in range(0,30):
          selection =  (SELECTIONmin +SELECTIONmax)/float(2)
```

```
        # test with new coef
        test = calcul(startSELECTION,stopSELECTION ,0,selection)
        # percentage obtained
        perX = test[0]
        # comparing obtained and expected
        if perX > perOBJ :
            SELECTIONmin = selection
        else :
            SELECTIONmax = selection
    print "start selection ",startSELECTION,"during", dureeSELECTION,"gen, selection coef=",selection

    if abs(float(perX - perOBJ)/perOBJ)>0.01:
        print " it is not possible to obtain the expected result"
        stop


    "--------------------------------------------"
    "CC) calculation of association for each centimorgan"
    for recombinaison in range (0,51):
        truc = calcul(startSELECTION,stopSELECTION ,recombinaison,selection)
        tout += truc[1]


"--------------------------------------------"

"DD) OUTPUT IN FILE "

ligne= ["distance","selection","startSEL","dureeSEL","generation", "taillePOP","selected", "neutral"]
tout2 = "\t".join(ligne)+"\n"+tout

fichier=open(dossier+"ModelSELECTION_"+model+typeSELECTION+".xls","w")
fichier.write(tout2)
fichier.close()
```
